# Cost-effectiveness analysis of ibrutinib plus venetoclax for relapsed or refractory mantle cell lymphoma in China and the United States

**DOI:** 10.3389/fpubh.2026.1817037

**Published:** 2026-05-21

**Authors:** Jing He, Xianxi Liu, Qing Yang

**Affiliations:** 1 School of Medicine, University of Electronic Science and Technology of China, Chengdu, China; 2 School of Nursing, Chengdu Medical College, Chengdu, China; 3 Department of Nursing, Sichuan Clinical Research Center for Cancer, Sichuan Cancer Hospital & Institute, Sichuan Cancer Center, University of Electronic Science and Technology of China, Chengdu, China

**Keywords:** ibrutinib, mantle cell lymphoma, partitioned survival model, cost-effectiveness, venetoclax

## Abstract

**Background:**

The SYMPATICO trial demonstrated that ibrutinib combined with venetoclax significantly improved progression-free survival in patients with relapsed or refractory (R/R) mantle cell lymphoma (MCL). This study evaluated the cost-effectiveness of the combination of ibrutinib–venetoclax for R/R MCL from the healthcare perspectives of China and the United States (US).

**Methods:**

A partitioned survival model with a 35-year simulation time horizon was developed to compare the cost-effectiveness of ibrutinib–venetoclax versus ibrutinib–placebo. Primary outcomes included total costs, life-years (LYs), quality-adjusted life-years (QALYs), and incremental cost-effectiveness ratios (ICERs). The robustness of the model was evaluated through one-way sensitivity analysis (OWSA) and probabilistic sensitivity analysis (PSA).

**Results:**

Base-case analysis showed that the ICER of ibrutinib–venetoclax versus ibrutinib–placebo was $128,183.93/QALY in China, which was significantly higher than the willingness-to-pay (WTP) threshold of $40,334/QALY. It was still not cost-effective below the $150,000/QALY WTP threshold in the US, with an ICER of $951,082.87/QALY. OWSA demonstrated the robustness of the model. PSA showed that ibrutinib–venetoclax had a 0% probability of being cost-effective under the current WTP thresholds in China and the US. Price reduction analysis indicated that in China, reducing the price of venetoclax to 20% of its original cost could achieve cost-effectiveness with a 60.80% probability. In the US, a combined price reduction of ibrutinib and venetoclax to 22.53% of their original costs is necessary to achieve a 50% probability of cost-effectiveness.

**Conclusion:**

Ibrutinib-venetoclax is unlikely to be cost-effective versus ibrutinib–placebo for R/R MCL in both China and the US.

## Introduction

In 2022, global cancer statistics reported approximately 553,000 new cases and 250,000 deaths from non-Hodgkin lymphoma (NHL), accounting for 2.8 and 2.6% of the worldwide cancer incidence and mortality, respectively (1). These figures place NHL among the top ten most common cancers and the eleventh leading cause of cancer-related death worldwide (2). Mantle cell lymphoma (MCL), a rare and aggressive subtype of NHL, comprises 2 to 6% of newly diagnosed NHL cases (3). Although chemotherapy achieves high overall response rates (ORR: 60–97%), the majority of MCL patients relapse within 18 months, resulting in a poor prognosis (4, 5). Patients who do not respond to initial treatment are classified as having relapsed/refractory MCL (R/R MCL) (6). In recent years, the advent of Bruton’s tyrosine kinase inhibitors (BTKi) and chimeric antigen receptor T-cell (CAR-T) therapies has expanded treatment options for R/R MCL (6). However, a standard treatment regimen remains elusive, and clinical management continues to face significant challenges (7). Existing studies highlight the substantial economic burden of MCL on patients and society. In China, the average annual total cost for patients with relapsed MCL reaches 480,000 yuan, with direct medical costs accounting for 81% (390,000 yuan) (8). Data from the United States (US) indicate that the average monthly expenses for second, third, and fourth-line treatments in R/R MCL patients are $29,999, $29,352, and $30,633, respectively, with BTKi treatment costs significantly increasing with each subsequent line of therapy (9).

Venetoclax, a BCL-2 inhibitor, received regulatory approval from the US Food and Drug Administration (FDA) in 2016 and the China National Medical Products Administration (NMPA) in 2020, and has since been included in medical insurance reimbursement programs. The recent SYMPATICO trial provided the first evidence that the combination of ibrutinib and venetoclax significantly extends progression-free survival (PFS) in patients with R/R MCL (median PFS: 31.9 months vs. 22.1 months; hazard ratio [HR] = 0.65, 95% confidence interval [CI]0.47–0.88, *p* = 0.0052). Additionally, overall survival (OS) data demonstrated a numerical advantage for the ibrutinib-venetoclax arm (median OS: 44.9 months; 95% CI 31.9- Not Estimable) versus the ibrutinib-placebo arm (median OS: 38.6 months; 95% CI 25.2–53.4), though this difference did not reach statistical significance (HR 0.85, 95% CI 0.61–1.19; *p* = 0.35) (10). While this regimen demonstrates significant survival benefits, its economic implications warrant critical evaluation. Indeed, despite the availability of novel agents such as BTK and BCL-2 inhibitors, high costs pose challenges to sustainable access, particularly in settings with constrained reimbursement frameworks (11).

To date, no economic evidence exists for the ibrutinib plus venetoclax regimen in the treatment of R/R MCL. This study aimed to construct a partitioned survival model (PSM) to evaluate the cost-effectiveness of ibrutinib plus venetoclax for the treatment of R/R MCL in China and the US, providing evidence-based support for clinical decision-making and the optimization of healthcare resource allocation.

## Materials and methods

This study adheres to the Consolidated Health Economic Evaluation Reporting Standards 2022 (CHEERS 2022) reporting guidelines (12) (Supplementary Table 1).

### Patients and intervention

The target population was consistent with the patient cohort of the SYMPATICO trial. Eligible participants were adults aged 18 and above, who had been pathologically diagnosed with R/R MCL after receiving one to five previous lines of therapy, and had an Eastern Cooperative Oncology Group (ECOG) performance status of 0–2. Patients were randomly assigned (1, 1) to receive oral ibrutinib 560 mg once daily, along with oral venetoclax (5-week ramp-up to 400 mg once daily) or placebo for 2 years, followed by single-agent ibrutinib 560 mg once daily until disease progression or unacceptable toxicity. Following disease progression, subsequent therapy was modeled based on trial data and guideline recommendations, assuming adoption of the bendamustine plus rituximab (BR) regimen, which involves administering bendamustine at a dose of 90 mg/m^2^ on days 2–3 and rituximab at a dose of 375 mg/m^2^ on day 1, with each 4-week period constituting one cycle. A maximum of six cycles is used, which complies with the Category 1 recommended treatment strategies for R/R MCL by both the National Comprehensive Cancer Network (NCCN) and Chinese Society of Clinical Oncology (CSCO) (13, 14). According to the SYMPATICO trial, 31% of patients in the venetoclax group and 45% in the placebo group received subsequent treatment. Therefore, based on these clinical observations and guideline consensus, the BR regimen was adopted as the reference standard for second-line therapy in our subsequent model design. The body surface area for Chinese and US patients was 1.72 m^2^ (15) and 1.96 m^2^ (16), respectively.

### Model overview

**Figure 1:**
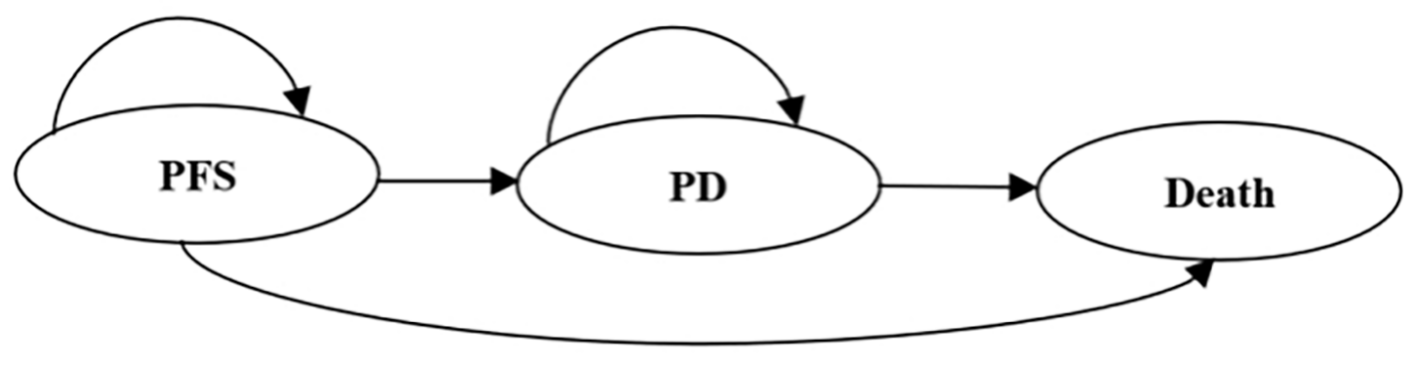
Structure of the partitioned survival model. PFS, progression-free survival; PD, progressive disease.

The PSM eliminates the need to calculate transition probabilities between states and avoids additional unnecessary modeling assumptions that could potentially affect the study outcomes, thus allowing for a more accurate simulation of disease occurrence and progression compared to the Markov model. A PSM was constructed using TreeAge Pro 2022, incorporating three mutually exclusive health states: PFS, progressive disease (PD), and death. All patients entered the model in the PFS state and could transition to different health states (Figure 1). The proportion of patients in the PFS state was directly derived from the PFS curve, the proportion of patients in the PD state was obtained by subtracting PFS from OS, and the proportion of patients in the death state was obtained by calculating 1 minus the OS curve. The cycle length was set to 1 month. Considering the median age of patients in the SYMPATICO trial was 68 years old, the simulation time horizon was set to 35 years. This time horizon was considered sufficient to capture lifetime clinical outcomes, as it extends well beyond the expected survival of the target population. With a median age of 68 years, a 35-year horizon encompasses a life expectancy of 103 years, which exceeds the upper bound of life expectancy in both China and the US, ensuring that nearly all patients are accounted for in the death state over the course of the simulation. This approach is consistent with guidelines for pharmacoeconomic evaluations recommending that time horizons should be long enough to capture all relevant differences in costs and outcomes between strategies (17). The primary model outputs were cumulative costs, life-years (LYs), quality-adjusted life-years (QALYs) and incremental cost-effectiveness ratios (ICERs).

According to the China Guidelines for Pharmacoeconomic Evaluation (17), the treatment regimen of ibrutinib plus venetoclax for R/R MCL was considered economically viable compared with ibrutinib plus placebo if the ICER was less than or equal to the willingness-to-pay (WTP) threshold; otherwise, it was deemed not cost-effective. The WTP threshold in China was set at three times the per capita Gross Domestic Product (GDP) in 2024, equivalent to $40,334/QALY, while the US WTP threshold was set at $150,000/QALY (18). Costs and health outcomes were discounted at annual rates of 5% in China (17) and 3% in the US (19).

### Clinical data

The clinical efficacy and safety data used in this study were derived from the SYMPATICO trial. Due to the unavailability of original individual patient data (IPD), we employed GetData Graph Digitizer version 2.26 to extract PFS and OS data points from Kaplan–Meier survival curves. Subsequently, the IPD were reconstructed using the method by Guyot et al. (20) with R software version 4.3.2 and the ‘survHE’ package. All seven parametric models (Exponential, Weibull, Gamma, Generalized Gamma, Gompertz, Log-normal, and Log-logistic) were fitted to each survival curve. A single best-fitting model was then selected for each endpoint and treatment arm independently based on the lowest AIC and BIC values, supplemented by visual inspection of the extrapolated curves for clinical plausibility (21, 22) (Supplementary Figures 1–3). The optimal distribution was selected based on the Akaike Information Criterion (AIC), Bayesian Information Criterion (BIC) (Supplementary Table 2), and visual inspection for clinical plausibility (23). The parameters of the best-fitting distributions are presented in Table 1.

**Table 1:** Model parameters inputs and the ranges of the sensitivity analysis.

Parameters	Baseline value	Range		Distribution	Reference
		Minimum	Maximum		
Clinical data					
Log-normal survival model of OS for Ibrutinib + Venetoclax group	meanlog = 3.827 sdlog = 1.727	–	–	Fixed	Model fitting
Log-normal survival model of PFS for Ibrutinib + Venetoclax group	meanlog = 3.501 sdlog = 1.598	–	–	Fixed	Model fitting
Log- logistic survival model of OS for Ibrutinib + Placebo group	shape = 1.012 scale = 38.350	–	–	Fixed	Model fitting
Log-normal survival model of PFS for Ibrutinib +Placebo group	meanlog = 2.960 sdlog = 1.512	–	–	Fixed	Model fitting
Costs of drugs in China (US$)					
Ibrutinib (140 mg)	22.07	17.66	26.48	Gamma	Yaozh.com
Venetoclax (100 mg)	38.46	30.77	46.15	Gamma	Yaozh.com
Rituximab (100 mg)	179.79	143.83	215.75	Gamma	Yaozh.com
Bendamustine (25 mg)	59.35	47.48	71.22	Gamma	Yaozh.com
Costs of drugs in the US (US$)					
Ibrutinib (560 mg)	879.20	703.36	1,055.04	Gamma	Red Book Online (24)
Venetoclax (20 mg)	26.00	20.8	31.2	Gamma	Red Book Online (24)
Rituximab (100 mg)	940.00	752	1,128	Gamma	Red Book Online (24)
Bendamustine (70 mg)	2,375.19	1,900.08	2,850.12	Gamma	Red Book Online (24)
Cost of disease management per cycle in China (US$)					
0–2 years	76.76	61.41	92.11	Gamma	(31)
3–5 years	44.63	35.70	53.56	Gamma	(31)
6 years and beyond	27.67	22.14	33.20	Gamma	(31)
Cost of disease management per cycle in the US (US$)					
PFS state	1,696.85	1,357.48	2,036.22	Gamma	(34)
PD state	8,435.78	6,748.62	10,122.94	Gamma	(34)
Cost of PET-CT per unit in China	736.52	589.22	883.82	Gamma	(31)
Cost of PET-CT per unit in the US	5,828.84	4,663.07	6,994.61	Gamma	(44)
Cost of end-of-life in China	5,289.31	4,231.45	6,347.17	Gamma	(27)
Cost of end-of-life in the US	28,163.46	22,530.77	33,796.15	Gamma	(30)
Cost of AEs in China (US$)					
Anemia	506.52	405.22	607.82	Gamma	(31)
Neutropenia	99.30	79.44	119.16	Gamma	(31)
Pneumonia	1,041.34	833.07	1,249.61	Gamma	(8)
Thrombocytopenia	161.41	129.13	193.69	Gamma	(31)
Leukopenia	220.37	176.30	264.44	Gamma	(28, 45)
Atrial fibrillation	645.75	516.60	774.90	Gamma	(28, 46)
Cost of AEs in the US (US$/admin)					
Anemia	8,234.58	6,587.66	9,881.50	Gamma	(34)
Neutropenia	12,176.13	9,740.90	14,611.36	Gamma	(34)
Pneumonia	20,113.76	16,091.01	24,136.51	Gamma	(34)
Thrombocytopenia	1,437.80	1,150.24	1,725.36	Gamma	(25)
Leukopenia	216.48	173.18	259.78	Gamma	(34)
Atrial fibrillation	22,541.81	18,033.45	27,050.17	Gamma	(34)
Risk of AEs in Ibrutinib + Placebo group					
Anemia	0.03	0.02	0.04	Beta	(10)
Neutropenia	0.11	0.09	0.13	Beta	(10)
Pneumonia	0.12	0.10	0.14	Beta	(10)
Thrombocytopenia	0.08	0.06	0.10	Beta	(10)
Leukopenia	0	-	-	Beta	(10)
Atrial fibrillation	0.05	0.04	0.06		(10)
Risk of AEs in Ibrutinib + Venetoclax group					
Anemia	0.10	0.08	0.12	Beta	(10)
Neutropenia	0.31	0.25	0.37	Beta	(10)
Pneumonia	0.11	0.09	0.13	Beta	(10)
Thrombocytopenia	0.13	0.10	0.16	Beta	(10)
Leukopenia	0.07	0.06	0.08	Beta	(10)
Atrial fibrillation	0.05	0.04	0.06	Beta	(10)
Proportion of patients received subsequent therapy					
Ibrutinib + Venetoclax group	0.31	0.25	0.37	Beta	(10)
Ibrutinib + Placebo group	0.45	0.36	0.54	Beta	(10)
Health utility					
PFS in Ibrutinib + Placebo group	0.824	0.767	0.867	Beta	(10)
PFS in Ibrutinib + Venetoclax group	0.827	0.755	0.86	Beta	(10)
PD	0.68	0.634	0.727	Beta	(32)
Anemia	0.007	0.006	0.008	Beta	(31)
Neutropenia	0.032	0.026	0.038	Beta	(31)
Pneumonia	0.150	0.120	0.180	Beta	(30)
Thrombocytopenia	0.038	0.030	0.046	Beta	(31)
Leukopenia	0.010	0.008	0.012	Beta	(31)
Atrial fibrillation	0.150	0.120	0.180	Beta	(31)
Body surface area (m^2^)					
China	1.72	1.38	2.06	Gamma	(15)
US	1.96	1.57	2.35	Gamma	(16)
Discount rate (%)					
China	5	0	8	Fixed	(17)
US	3	0	8	Fixed	(19)

### Costs and utilities

In this study, only direct medical costs were considered from the perspective of the healthcare systems in China and the US, with costs in the US specifically based on Medicare reimbursement rates, including costs associated with drug acquisition, disease management, Positron Emission Tomography-Computed Tomography (PET-CT) scans, management of severe adverse events (AEs), and end-of-life care, and subsequent treatment. Drug costs in China were determined based on the average winning bid prices from the YaoZH database^1^ in 2024; the drug costs in the US were sourced from Red Book Online (24), utilizing the average wholesale acquisition cost. Other cost data were derived from published literature (15, 25–29). Since the SYMPATICO trial only reported the overall incidence rates of severe AEs, the model was unable to accurately simulate the timing of these AEs and the associated costs. We referred to similar studies (30, 31) and calculated the total cost of AEs by multiplying the incidence rate of each AE by its treatment cost, incorporating the total AE cost into the first cycle. Consistent with established pharmacoeconomic conventions and previous cost-effectiveness analyses in oncology, only grade 3 or higher AEs with an incidence rate exceeding 5% in either treatment arm were included in the model. This threshold was selected because grade 1–2 AEs are typically manageable with minimal or no additional medical intervention and are unlikely to substantially impact healthcare resource utilization or total costs. Severe AEs (grade ≥3), by contrast, often require hospitalization, dose modifications, treatment discontinuation, or specialist management, thereby exerting a more significant economic burden. The 5% incidence cutoff was applied to exclude rare events that would have negligible impact on overall cost estimates while retaining clinically relevant toxicities. All costs were adjusted to the 2024 price level using the local consumer price index (CPI) based on formulas from https://www.inflationtool.com and converted to US dollars (2024 annual average exchange rate, $1 = RMB 7.1217).

Utility values for PFS were derived from longitudinal EuroQoL 5 Dimensions 5 Levels (EQ-5D-5L) measurements in the SYMPATICO trial, while utilities for PD were sourced from published literature due to the absence of PD-specific quality-of-life assessments in the trial (32). Additionally, we accounted for decreases in utility values due to grade 3 or higher AEs with an incidence rate greater than 5%. Similarly, the disutility associated with AEs was only considered in the first cycle. All costs and utilities are presented in Table 1.

### Scenario analyses

We conducted four scenario analyses. First, shorter time horizons of 10 and 20 years were evaluated to assess the impact on model outcomes. Additionally, we assumed the price reductions for venetoclax at various levels (80, 60, 40, and 20% of the original price) to further explore the cost-effectiveness of the ibrutinib plus venetoclax regimen. Third, to assess the uncertainty in model outputs due to the choice of survival models, we employed flexible survival models, including fractional polynomial models, restricted cubic spline models, and Royston-Parmar (RP) spline models (33) (Supplementary Tables 3, 4 and Supplementary Figure 4, 5). Finally, to further evaluate the robustness of our findings to alternative utility assumptions, we conducted a scenario analysis incorporating utility values of 0.843 for the PFS state and 0.743 for the PD state, as reported in the US cost-effectiveness analysis of R/R MCL by Simons et al. (34).

### Sensitivity analyses

The robustness of model outputs was assessed through one-way sensitivity analysis (OWSA) and probabilistic sensitivity analysis (PSA). We conducted OWSA to examine the robustness of the ICER. The range of parameter variation was determined based on 95% confidence intervals (CIs) reported in the literature or ±20% of the baseline value. Tornado diagrams were used to visualize the impact of parameter uncertainty. For the PSA, all parameters were sampled from predefined statistical distributions, with 1,000 Monte Carlo simulations performed. Costs and body surface area were assumed to follow a gamma distribution, while utility values and probabilities were assumed to follow a beta distribution Distribution parameters were estimated from the mean and standard deviation (SD) of each parameter. The SD was derived as the 95% CI width divided by 3.92 when reported. For parameters varied by ±20% of the base‑case value, the SD was approximated as 10% of the base-case value. For the beta distribution, the shape parameters were calculated as α = mean(mean(1−mean)/SD² − 1) and β = (1−mean)(mean(1−mean)/SD² − 1). For the gamma distribution, the shape and rate parameters were calculated as α = mean²/SD² and λ = mean/SD², respectively. The results were displayed as an incremental cost-effectiveness scatter plot and a cost-effectiveness acceptability curve.

## Results

### Base-case analysis results

In China, the total cost for the ibrutinib plus venetoclax group was $131,805.05, with 5.73 LYs and 4.53 QALYs gained, while the total cost for the ibrutinib plus placebo group was $48,991.09, with 5.10 LYs and 3.89 QALYs. The ICER was $128,183.93/QALY, which is significantly higher than China’s WTP threshold. In the US, the total cost for the ibrutinib plus venetoclax group was $2,239,383.62, with 6.72 LYs and 5.31 QALYs, while the total cost for the ibrutinib plus placebo group was $1,468,076.34, with 5.92 LYs and 4.50 QALYs. The ICER was $951,082.87/QALY, exceeding the US WTP threshold of $150,000/QALY. Therefore, ibrutinib plus venetoclax was not cost-effective in either China or the US. The base-case analysis results are shown in Table 2.

**Table 2:** Results of the base-case and scenario analyses.

Strategies and scenarios	Chinese					US				
	Cost ($)	LYs	QALYs	ICER ($/QALY)	Acceptability	Cost ($)	LYs	QALYs	ICER ($/QALY)	Acceptability
Base-case analysis										
Ibrutinib + placebo	48,991.09	5.10	3.89	–	–	1,468,076.34	5.92	4.50	–	–
Ibrutinib + venetoclax	131,805.05	5.73	4.53	128,183.93	0%	2,239,383.62	6.72	5.31	951,082.87	0%
Employing flexible parametric models										
Ibrutinib + placebo	50,968.51	5.13	3.83	–	–	1,294,342.82	5.97	4.42	–	–
Ibrutinib + venetoclax	130,572.37	5.78	4.58	106,575.19	0%	2,262,274.27	6.81	5.38	1,008,971.03	0%
Using alternative utility values										
Ibrutinib + placebo	48,991.09	5.10	4.07	–	–	1,468,076.34	5.92	4.72	–	–
Ibrutinib + venetoclax	131,805.05	5.73	4.68	136,598.35	0%	2,239,383.62	6.72	5.48	1,007,870.47	0%
10-time horizon										
Ibrutinib + placebo	47,716.99	3.79	2.92	–	–	1,109,281.87	4.05	3.13	–	–
Ibrutinib + venetoclax	130,261.12	4.11	3.27	235,943.77	0%	1,643,824.63	4.41	3.51	1,380,016.94	0%
20-time horizon										
Ibrutinib + placebo	48,592.02	4.67	3.58	–	–	1,343,483.99	5.21	3.98	–	–
Ibrutinib + venetoclax	131,257.17	5.19	4.12	151,427.20	0%	2,022,640.82	5.84	4.63	1,051,879.72	0%
80% price of venetoclax										
Ibrutinib + venetoclax	116,454.27	5.73	4.53	104,423.16	0%	2,187,131.45	6.72	5.31	886,651.81	0%
60% price of venetoclax										
Ibrutinib + venetoclax	101,103.49	5.73	4.53	80,662.40	7%	2,134,879.28	6.72	5.31	822,220.75	0%
40% price of venetoclax										
Ibrutinib + venetoclax	85,752.71	5.73	4.53	56,901.63	29.30%	2,082,627.11	6.72	5.31	757,789.70	0%
20% price of venetoclax										
Ibrutinib + venetoclax	70,401.94	5.73	4.53	33,140.86	60.80%	2,030,374.94	6.72	5.31	693,358.64	0%

LYs, life-years; QALYs, quality-adjusted life-years; ICER, incremental cost-effectiveness ratio.

### Scenario analyses

In the scenario analysis, as the simulation time horizon shortened, the ICER values increased. In the context of China, reducing the price of venetoclax to 20% of its current level ($7.692 per 100 mg) lowered the ICER to $33,140.86 per QALY, which is below China’s WTP threshold. However, for the US, despite the reduction in ICER due to price cuts of venetoclax, the cost-effectiveness was still not achieved. Besides, using RP spline models compared to standard parametric models, the ICERs decreased from the Chinese perspective, but increased from the US perspective. Overall, the flexible survival models demonstrated consistent findings with the base-case analysis. Under the scenario with alternative utility values, the analysis yielded ICERs of $1,007,870.47/QALY and $136,598.35/QALY from the US and Chinese healthcare perspectives, respectively. Both estimates remained markedly above the respective country-specific WTP thresholds, further corroborating the robustness of the base-case conclusions. Detailed results are presented in Table 2.

### Sensitivity analyses

**Figure 2:**
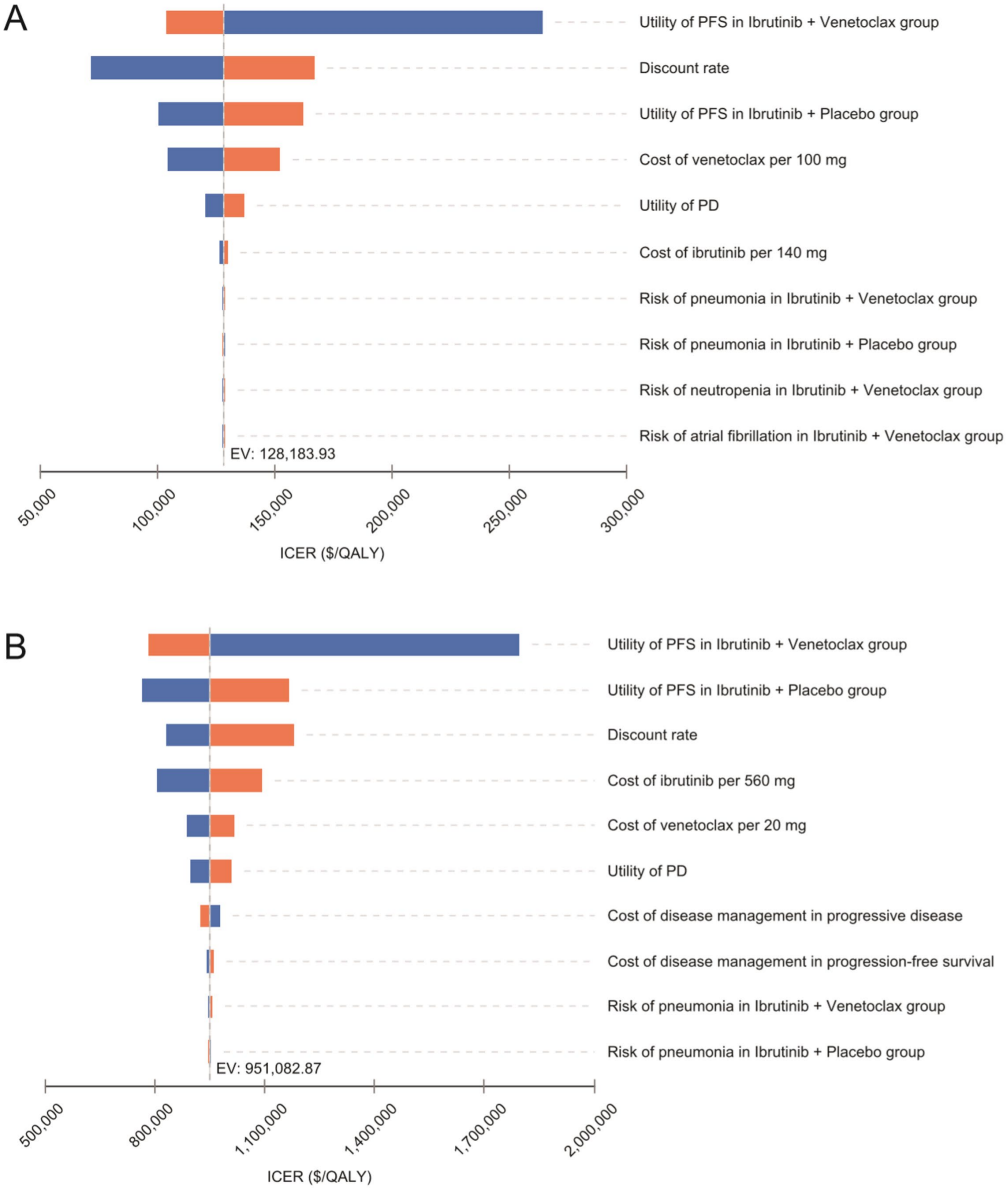
Tornado diagram of one-way sensitivity analysis of ibrutinib plus venetoclax versus ibrutinib plus placebo. **(A)** China setting. **(B)** US setting. ICER, incremental cost-effectiveness ratio; QALY, quality-adjusted life year; PFS, progression-free survival; PD, progressive disease.

The tornado diagram of the OWSA (Figure 2) showed that, from the Chinese perspective, utility of PFS of the two treatment groups, discount rate, and cost of venetoclax had the most significant impact on the ICER. In the context of the US, the most sensitive factors to the ICER were utility of PFS, discount rate, and cost of ibrutinib and venetoclax. Other parameters slightly affected the ICERs, but none could reduce the ICER below the threshold.

**Figure 3:**
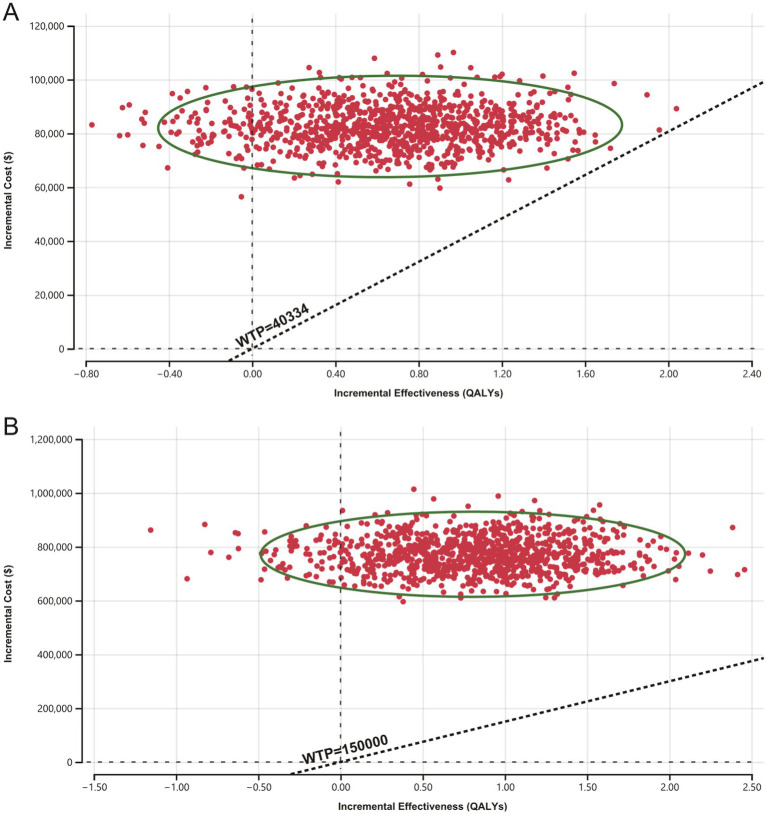
Incremental cost-effectiveness scatter plot of ibrutinib plus venetoclax versus ibrutinib plus placebo. **(A)** China setting. **(B)** US setting. WTP, willingness-to-pay; QALYs, quality-adjusted life years.

**Figure 4:**
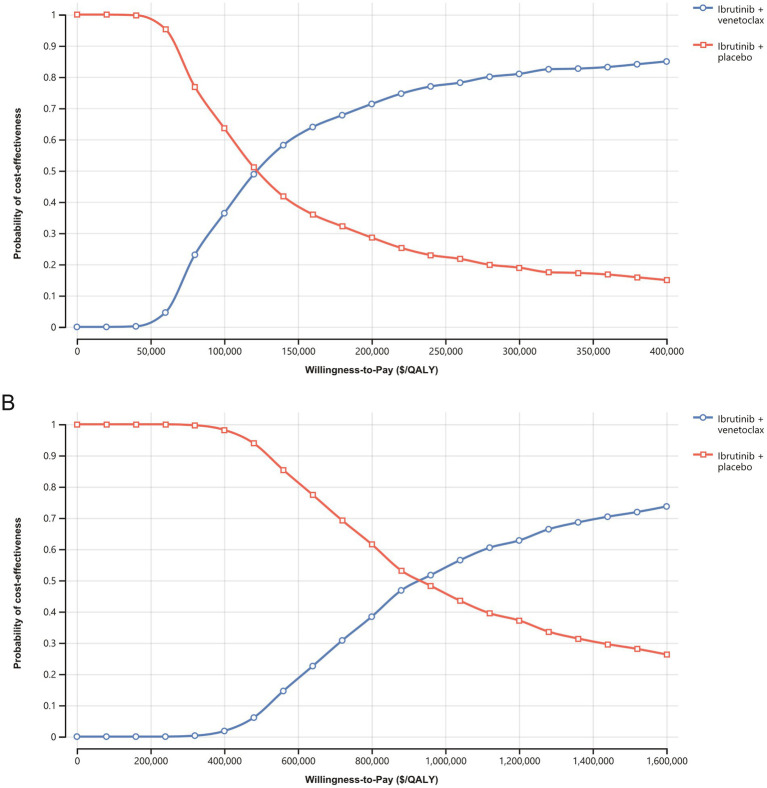
Cost-effectiveness acceptability curve of ibrutinib plus venetoclax versus ibrutinib plus placebo. **(A)** China setting. **(B)** US setting. WTP, willingness-to-pay; QALY, quality-adjusted life year.

The incremental cost-effectiveness scatter plots (Figure 3) for both countries show that all ICER scatter points were located in the first quadrant of the incremental cost-effectiveness plane, concentrated within the 95% CI, and above the WTP threshold line. The cost-effectiveness acceptability curves (Figure 4) indicated that, for both China and the US, the probability of ibrutinib plus venetoclax being cost-effective at the WTP thresholds set in this study is 0%. However, the combination regimen would only be considered cost‑effective at its current price if the WTP thresholds in China and the US were substantially raised to approximately $122,000/QALY and $896,000/QALY, respectively. In China, a price reduction of venetoclax to 60% of its original cost has a 29.30% probability of being cost-effective, while a reduction to 20% of the original price increases this probability to 60.80% (Table 2). In contrast, in the US, even reducing the price of venetoclax to 20% of its original level remains not cost-effective (Table 2). Further exploratory analysis found that when the prices of ibrutinib and venetoclax were both reduced to 22.53% of their current costs, the probability of the ibrutinib plus venetoclax strategy being cost-effective reached 50%.

## Discussion

To our knowledge, Shi et al. (31) demonstrated the superior cost-effectiveness of zanubrutinib within China’s healthcare setting. Meanwhile, studies by Alrawashdh et al. (25) and Simons et al. (34) confirmed the economic benefits of second-generation BTK inhibitors and CAR-T therapy, respectively, in R/R MCL. Nevertheless, although several studies have evaluated the cost-effectiveness of various lymphoma treatment regimens, a formal cost-effectiveness analysis of the ibrutinib-venetoclax combination remains lacking. This is the first study to model the economic evaluation of ibrutinib plus venetoclax for treating R/R MCL from the perspectives of the healthcare systems in China and the US. Our results indicated that compared with ibrutinib plus placebo, the ibrutinib plus venetoclax regimen gained more LYs and QALYs, but at a higher cost. The ICERs were $128,183.93/QALY in China and $951,082.87/QALY in the US, both of which significantly exceed the current WTP thresholds in the two countries. Therefore, this combination therapy cannot be considered a cost-effective option. Sensitivity analyses confirmed the robustness of the results.

Scenario analysis demonstrates that reducing the price of venetoclax to 20% of its original cost in China is highly likely to achieve cost-effectiveness, with a probability of 60.80%. Such a price reduction is feasible through mechanisms including China’s National Reimbursement Drug List (NRDL) negotiations or volume-based procurement (35, 36). However, in the US, cost-effectiveness remains unattainable even with the maximum possible price reductions for venetoclax. PSA showed that the combination therapy of ibrutinib and venetoclax would only achieve cost-effectiveness in 50% of scenarios if the prices of both drugs are reduced to 22.53% of their original costs. This economic infeasibility likely stems from structural differences in pricing mechanisms, with the centralized price negotiation absent in the US healthcare system compared to the bargaining power of China’s NRDL (37). Under the current US policy environment, price reductions for venetoclax and ibrutinib primarily rely on general policy mechanisms, with a lack of specialized measures targeting their combination therapy. Ibrutinib, selected in the first round of Medicare Part D price negotiations, will be subject to the “Maximum Fair Price” (MFP) mechanism, which is expected to reduce federal Medicare spending, though the negotiations do not directly address combination therapy scenarios (38). Price reductions for venetoclax depend more on the Medicaid Drug Rebate Program (MDRP), the 340B Drug Pricing Program, pharmaceutical manufacturer patient assistance programs (PAPs), and potential future outcomes-based reimbursement agreements (39). At present, there are no federal price reduction programs specifically aimed at R/R MCL or the ibrutinib-venetoclax combination therapy; its cost optimization relies solely on the combined effects of the aforementioned general leverage mechanisms (40).

The OWSA identified utility of PFS as the parameter with the largest impact on model outcomes; however, sensitivity variations within plausible ranges did not reverse the conclusion that ibrutinib plus venetoclax is not cost-effective at current prices. Scenario analyses applying alternative PFS utility values further reinforced the robustness of the base-case findings. Moreover, the cost of venetoclax was found to be critical determinant of the ICER in the Chinese setting, highlighting that venetoclax pricing is a central concern in national reimbursement decision-making. In contrast, in the US, the costs of ibrutinib and venetoclax are the primary driver affecting the ICER, which implies that cost control for both ibrutinib and venetoclax needs to be considered in price negotiations. This difference points to the need for different strategies in drug price negotiations in the two countries to accommodate their respective healthcare systems and economic conditions. We recommend healthcare authorities prioritize targeted pricing interventions through NRDL negotiations or volume-based procurement (35, 36). Even an 80% price reduction for venetoclax fails to achieve cost-effectiveness. Legislative action to establish centralized bargaining mechanisms is imperative to overcome systemic cost barriers for dual-agent therapies. Although the synergistic mechanism between ibrutinib (41, 42) and venetoclax enhances therapeutic efficacy, cost constraints limit their accessibility.

Despite significantly improved PFS, the SYMPATICO trial found no statistically significant OS benefit for venetoclax plus ibrutinib versus ibrutinib alone. As our model relies on OS and PFS curve extrapolation, this likely resulted in a smaller QALY gain for the combination regimen. Consequently, the high cost of venetoclax relative to the limited health outcome improvement precluded cost-effectiveness. Additionally, to address the potential limitations of standard parametric survival models in capturing the shape of the hazard function, we utilized flexible survival models to mitigate model selection bias (43). The results indicated that although there were changes in the ICER, the minor variations were not significant enough to alter the conclusions drawn.

This study has some limitations. First, based on the SYMPATICO study, the study cohort mainly included white participants, with less than 5% of Asian participants, which may limit its applicability to Chinese patients. Ethnic differences in drug metabolism, particularly variations in cytochrome P450 enzyme activity, may affect the pharmacokinetics of both ibrutinib and venetoclax. However, given the lack of head-to-head efficacy data specifically in Asian or Chinese populations, extrapolating the survival benefits from the predominantly white SYMPATICO cohort to Chinese patients remains a necessary but cautious assumption. Future studies incorporating Asian patient data are warranted to validate these findings. Second, as the SYMPATICO trial did not report health state utilities for PD, these values were derived from published literature. However, heterogeneity in treatment regimens and study populations across source literature may introduce bias in QALY estimates. Additionally, the original study did not specify which EQ-5D-5L value set was applied, and no domestically derived utility value study has been published for the Chinese population in R/R MCL. Consequently, the model could not differentiate utility values between China and the US, and the same values were uniformly applied to both countries. This approach may not fully capture population-specific preferences in health state valuation. Nevertheless, scenario analyses demonstrated that utility uncertainty did not materially affect the conclusions. Third, the OS data reported in the SYMPATICO study are not mature, and extrapolating long-term survival curves based on limited follow-up data using standard parametric distributions will inevitably introduce uncertainty into model outputs. However, this is an inherent flaw in the methodology, and the results of this study can be further verified with updated survival data in the future. Fourth, the assumption that all patients received the BR regimen upon disease progression represents a necessary modeling simplification; we assumed that the BR regimen would be used after disease progression, which may differ from actual treatment choices. Fifth, our model only included the management costs and disutilities related to AEs of grade 3 or higher with an incidence rate greater than 5%, following conventions in prior economic evaluations. While this approach may underestimate the total costs of the model and overestimate QALYs to some extent, the OWSA showed that AE-related parameters had a minor impact on the model results and were unlikely to reverse the conclusions.

## Conclusion

In summary, from the perspective of the healthcare systems in both China and the US, ibrutinib plus venetoclax is unlikely to be cost-effective for the treatment of R/R MCL compared with ibrutinib alone. Reducing the cost of venetoclax in China or simultaneously lowering the costs of both venetoclax and ibrutinib in the US may improve their cost-effectiveness. However, recognizing the limitations of the study methods, it emphasizes the need for additional high-quality real-world data. Future research should prioritize obtaining long-term follow-up and real-world data to validate the robustness of the model outcomes.

## Data Availability

The original contributions presented in the study are included in the article/Supplementary material, further inquiries can be directed to the corresponding author.

## Footnotes

1
https://data.yaozh.com/
