# Correction: Cost-effectiveness analysis of ibrutinib plus venetoclax for relapsed or refractory mantle cell lymphoma in China and the United States

**DOI:** 10.3389/fpubh.2026.1894932

**Published:** 2026-06-29

**Authors:** Jing He, Xianxi Liu, Qing Yang

**Affiliations:** 1 School of Medicine, University of Electronic Science and Technology of China, Chengdu, China; 2 School of Nursing, Chengdu Medical College, Chengdu, China; 3 Department of Nursing, Sichuan Clinical Research Center for Cancer, Sichuan Cancer Hospital & Institute, Sichuan Cancer Center, University of Electronic Science and Technology of China, Chengdu, China

**Keywords:** ibrutinib, mantle cell lymphoma, partitioned survival model, cost-effectiveness, venetoclax

Author Qing Yang was erroneously assigned to affiliation 1. This affiliation has now been removed for author Qing Yang. The correct author affiliation for Qing Yang is affiliation 3.

There was a mistake in Figures 2–4 as published. The corrected Figures 2–4 are appears below.

**Figure 2:**
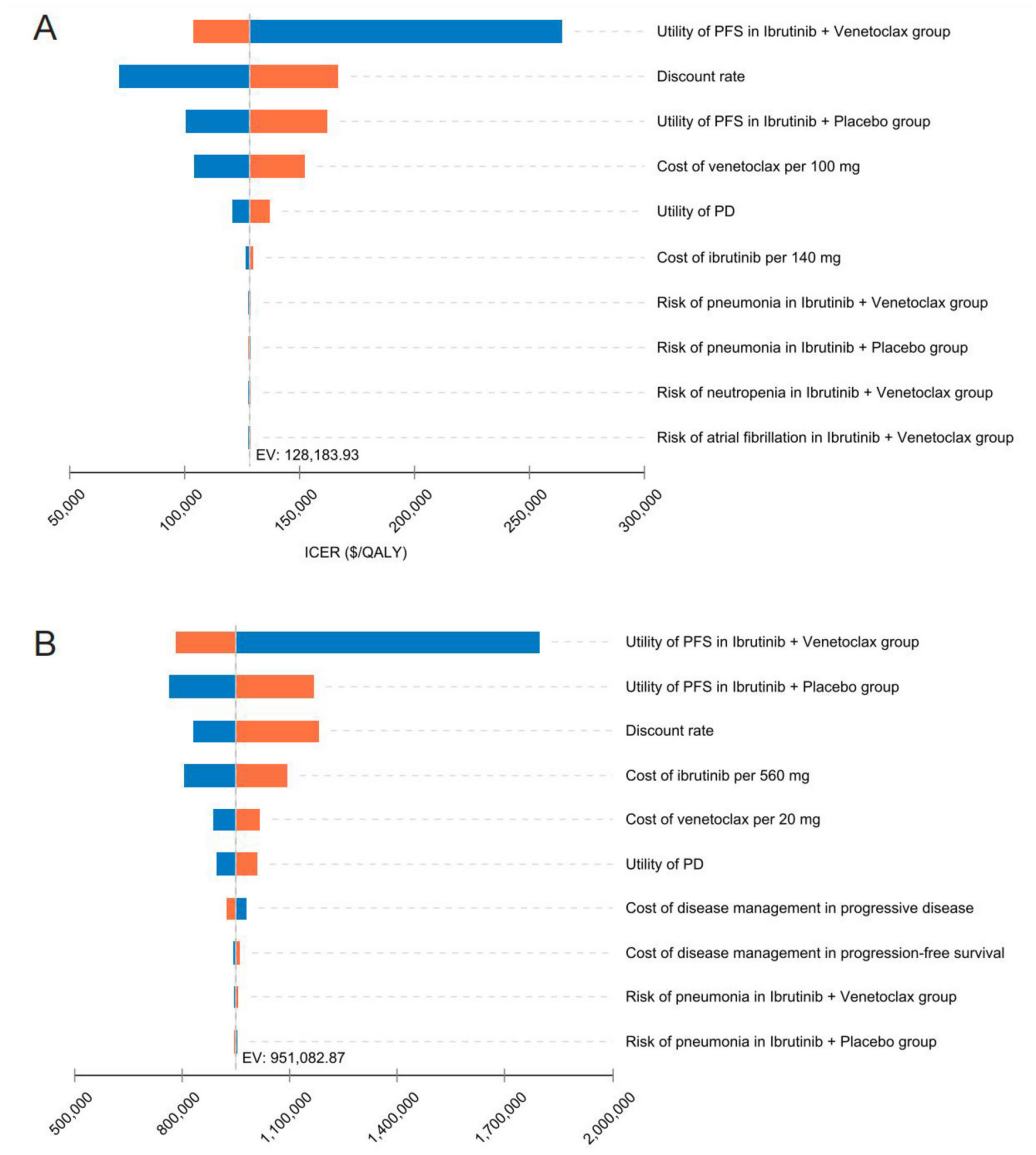
Tornado diagram of one-way sensitivity analysis of ibrutinib plus venetoclax versus ibrutinib plus placebo. **(A)** China setting. **(B)** US setting. ICER, incremental cost-effectiveness ratio; QALY, quality-adjusted life year; PFS, progression-free survival; PD, progressive disease.

**Figure 3:**
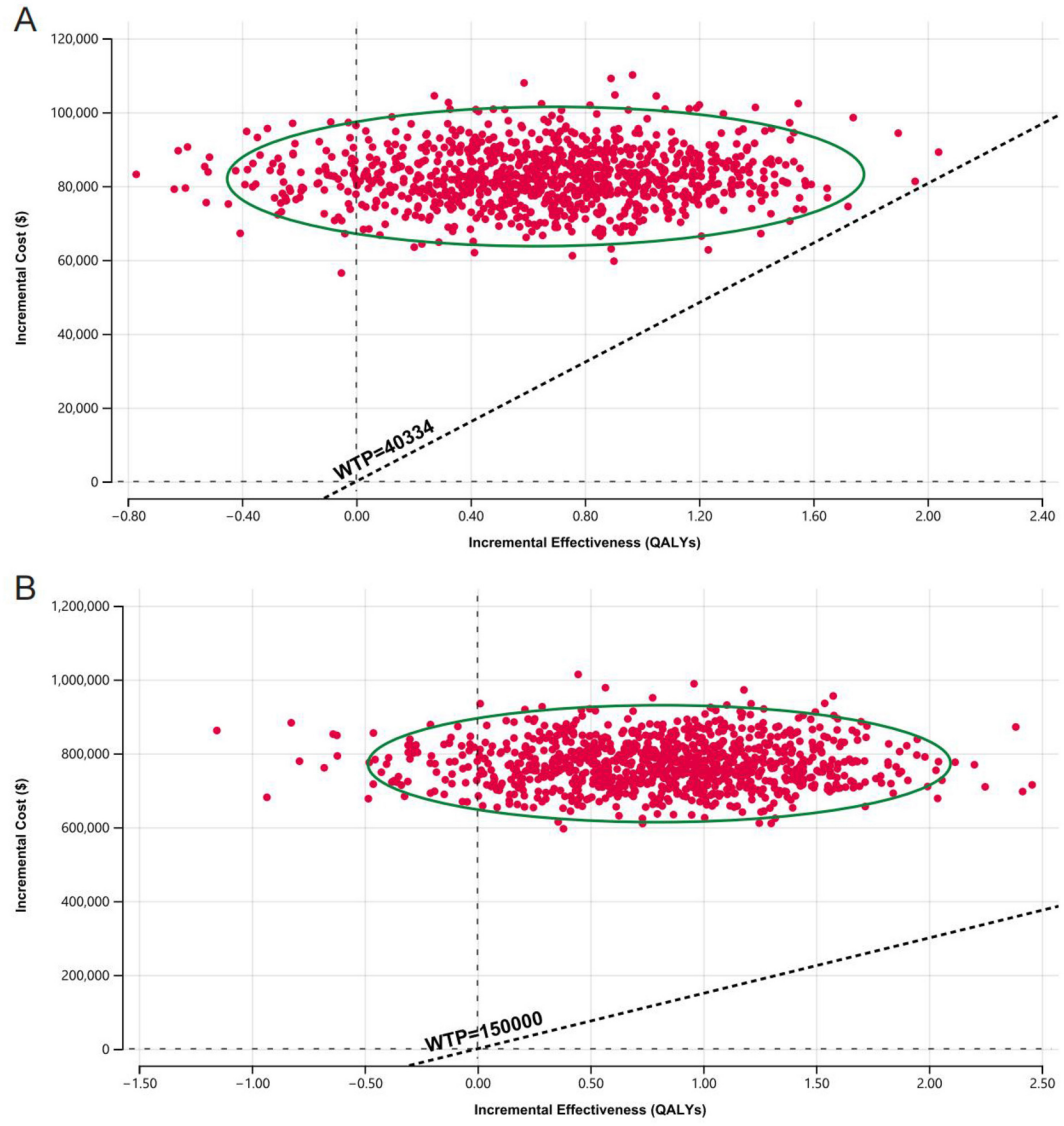
Incremental cost-effectiveness scatter plot of ibrutinib plus venetoclax versus ibrutinib plus placebo. **(A)** China setting. **(B)** US setting. WTP, willingness-to-pay; QALYs, quality-adjusted life years.

**Figure 4:**
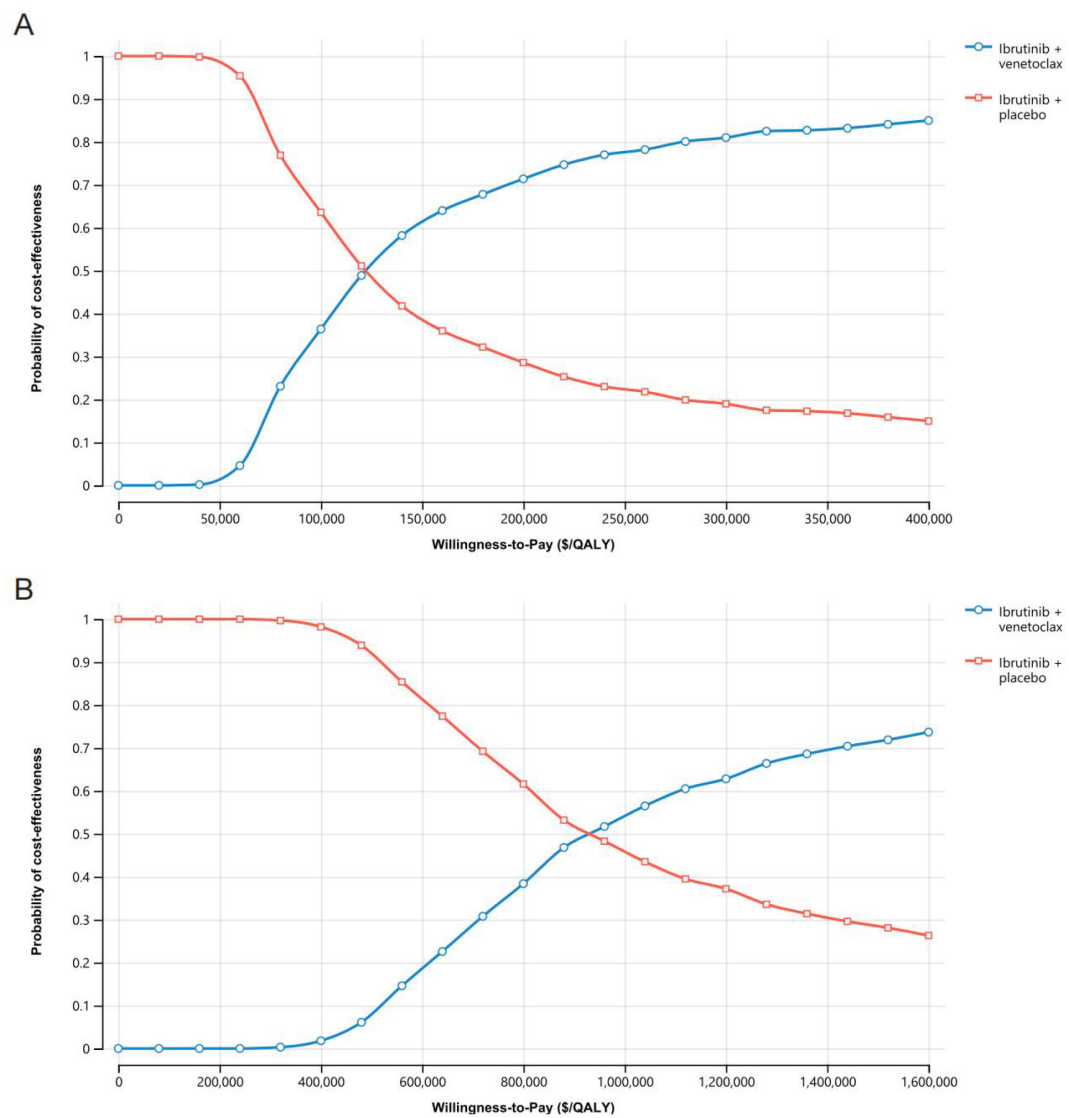
Cost-effectiveness acceptability curve of ibrutinib plus venetoclax versus ibrutinib plus placebo. **(A)** China setting. **(B)** US setting. WTP, willingness-to-pay; QALY, quality-adjusted life year.

The original version of this article has been updated.

