# Supplementary material for: Cost-effectiveness analysis of ibrutinib plus venetoclax for relapsed or refractory mantle cell lymphoma in China and the United States
Source: Front Public Health. 2026 May 21;14:1817037. doi: 10.3389/fpubh.2026.1817037 (PMC13233502; doi:10.3389/fpubh.2026.1817037)
Supplement: Supplementary file 1 [file Data_Sheet_1.docx]

Supplementary Material

# Supplementary Tables

**Supplementary Table 1.** CHEERS Checklist 2022.

| **Topic** | **No** | **Item** | **Reported** |
| --- | --- | --- | --- |
| **Title** | | | |
| Title | 1 | Identify the study as an economic evaluation and specify the  interventions being compared | Yes |
| **Abstract** | | | |
| Abstract | 2 | Provide a structured summary that highlights context, key  methods, results, and alternative analyses | Yes |
| **Introduction** | | | |
| Background and objectives | 3 | Give the context for the study, the study question, and its  practical relevance for decision making in policy or practice | Yes |
| **Methods** | | | |
| Health economic analysis plan | 4 | Indicate whether a health economic analysis plan was developed  and where available | Yes |
| Study population | 5 | Describe characteristics of the study population (such as age  range, demographics, socioeconomic, or clinical characteristics) | Yes |
| Setting and location | 6 | Provide relevant contextual information that may influence  findings | Yes |
| Comparators | 7 | Describe the interventions or strategies being compared and why  chosen | Yes |
| Perspective | 8 | State the perspective(s) adopted by the study and why chosen | Yes |
| Time horizon | 9 | State the time horizon for the study and why appropriate | Yes |
| Discount rate | 10 | Report the discount rate(s) and reason chosen | Yes |
| Selection of outcomes | 11 | Describe what outcomes were used as the measure(s) of  benefit(s) and harm(s) | Yes |
| Measurement of outcomes | 12 | Describe how outcomes used to capture benefit(s) and harm(s)  were measured | Yes |
| Valuation of outcomes | 13 | Describe the population and methods used to measure and value  outcomes | Yes |
| Measurement and valuation of  resources and costs | 14 | Describe how costs were valued | Yes |
| Currency, price date, and  conversion | 15 | Report the dates of the estimated resource quantities and unit  costs, plus the currency and year of conversion | Yes |
| Rationale and description of model | 16 | If modelling is used, describe in detail and why used. Report if  the model is publicly available and where it can be accessed | Yes |
| Analytics and assumptions | 17 | Describe any methods for analysing or statistically transforming  data, any extrapolation methods, and approaches for validating | Yes |
|  |  | any model used |  |
| Characterising heterogeneity | 18 | Describe any methods used for estimating how the results of the  study vary for subgroups | Yes |
| Characterising distributional  effects | 19 | Describe how impacts are distributed across different individuals  or adjustments made to reflect priority populations | Yes |
| Characterising uncertainty | 20 | Describe methods to characterise any sources of uncertainty in  the analysis | Yes |
| Approach to engagement with  patients and others affected by the study | 21 | Describe any approaches to engage patients or service recipients,  the general public, communities, or stakeholders (such as clinicians or payers) in the design of the study | Not  applicable |
| **Results** |  |  |  |
| Study parameters | 22 | Report all analytic inputs (such as values, ranges, references)  including uncertainty or distributional assumptions | Yes |
| Summary of main results | 23 | Report the mean values for the main categories of costs and  outcomes of interest and summarise them in the most appropriate overall measure | Yes |
| Effect of uncertainty | 24 | Describe how uncertainty about analytic judgments, inputs, or projections affect findings. Report the effect of choice of  discount rate and time horizon, if applicable | Yes |
| Effect of engagement with patients and others affected by the study | 25 | Report on any difference patient/service recipient, general public, community, or stakeholder involvement made to the  approach or findings of the study | Not  applicable |
| **Discussion** |  |  |  |
| Study findings, limitations, generalisability, and current  knowledge | 26 | Report key findings, limitations, ethical or equity considerations not captured, and how these could affect patients, policy, or  practice | Yes |
| Other relevant information Source  of funding | 27 | Describe how the study was funded and any role of the funder in  the identification, design, conduct, and reporting of the analysis | Yes |
| Conflicts of interest | 28 | Report authors conflicts of interest according to journal or  International Committee of Medical Journal Editors requirements | Yes |

**Supplementary Table 2.** Summary of the statistical goodness-of-fit of Kaplan-Meier survival curves

|  | **Exponential** | **Weibull** | **Gamma** | **Generalized gamma** | **Gompertz** | **Log-normal** | **Log-logistic** |
| --- | --- | --- | --- | --- | --- | --- | --- |
| Ibrutinib + venetoclax group OS curve | | | | | | | |
| AIC | 715.8539 | 715.6037 | 716.1520 | 714.4378 | 713.4920 | 712.4733 | 713.4901 |
| BIC | 718.7517 | 721.3994 | 721.9476 | 723.1313 | 719.2877 | 718.2689 | 719.2858 |
| Ibrutinib + placebo group OS curve | | | | | | | |
| AIC | 758.6160 | 757.4881 | 758.0117 | 758.0060 | 756.0324 | 756.8526 | 756.0782 |
| BIC | 761.5064 | 763.2688 | 763.7924 | 766.6770 | 761.8131 | 762.6333 | 761.8589 |
| Ibrutinib + venetoclax group PFS curve | | | | | | | |
| AIC | 733.3022 | 732.596 | 733.6025 | 723.8122 | 727.6212 | 724.0816 | 727.8897 |
| BIC | 736.200 | 738.3917 | 739.3982 | 732.5058 | 733.4169 | 729.8773 | 733.6854 |
| Ibrutinib + placebo group PFS curve | | | | | | | |
| AIC | 838.5352 | 838.3681 | 838.9427 | 837.633 | 837.6787 | 836.0895 | 837.7915 |
| BIC | 841.4256 | 844.1488 | 844.7234 | 846.304 | 843.4594 | 841.8702 | 843.5722 |

**Supplementary Table 3.** Goodness-of-fit results of flexible survival models.

|  | Venetoclax-PFS | | | | Venetoclax-OS | | | Ibrutinib-PFS | | | Ibrutinib-OS | | |
| --- | --- | --- | --- | --- | --- | --- | --- | --- | --- | --- | --- | --- | --- |
| Model | Ln L | Params | | AIC | Ln L | Params | AIC | Ln L | Params | AIC | Ln L | Params | AIC |
| FP1-1 | -73.37 | | 2 | 150.73 | -74.49 | 2 | 152.99 | -92.85 | 2 | 189.70 | -75.87 | 2 | 155.73 |
| FP1-2 | -74.14 | | 2 | 152.28 | -77.52 | 2 | 159.03 | -92.36 | 2 | 188.72 | -76.61 | 2 | 157.21 |
| FP2-1 | -72.43 | | 3 | 150.87 | -74.07 | 3 | 154.13 | -91.90 | 3 | 189.80 | -75.45 | 3 | 156.91 |
| FP2-2 | -72.43 | | 3 | 150.87 | -74.11 | 3 | 154.23 | -91.76 | 3 | 189.52 | -75.46 | 3 | 156.91 |
| RCS1 | -72.42 | | 3 | 150.84 | -74.16 | 3 | 154.31 | -91.66 | 3 | 189.31 | -75.45 | 3 | 156.91 |
| RCS2 | -72.42 | | 4 | 152.84 | -74.14 | 4 | 156.28 | -91.24 | 4 | 190.49 | -75.45 | 4 | 158.91 |
| RP-hazard-1 | -72.23 | | 3 | 150.47 | -73.23 | 4 | 154.47 | -90.75 | 4 | 189.49 | -76.02 | 2 | 156.04 |
| RP-hazard-2 | -73.29 | | 2 | 150.58 | -73.59 | 3 | 153.17 | -91.43 | 2 | 186.86 | -75.77 | 3 | 157.54 |
| RP-odds-1 | -72.58 | | 2 | 149.16 | -73.05 | 4 | 154.10 | -90.52 | 4 | 189.05 | -75.56 | 2 | 155.11 |
| RP-odds-2 | -72.29 | | 3 | 150.58 | -73.52 | 3 | 153.04 | -92.13 | 2 | 188.25 | -75.57 | 3 | 157.13 |
| RP-normal-1 | -72.39 | | 2 | 148.79 | -73.51 | 2 | 151.02 | -90.62 | 4 | 189.23 | -76.23 | 2 | 156.45 |
| RP-normal-2 | -72.40 | | 3 | 150.79 | -73.41 | 3 | 152.82 | -92.34 | 2 | 188.68 | -75.61 | 3 | 157.21 |

Abbreviations: OS: overall survival; PFS: progression-free survival; LnL: log likelihood; Params: Parameters; AIC: Akaike information criterion; FP: fractional polynomial; RCS: restricted cubic spline models; RP: Royston-Parmar models.

**Supplementary Table 4.** Clinical efficacy parameters of flexible survival models.

| **Group** | **Model** | **Parameters** |
| --- | --- | --- |
| OS of Ibrutinib + venetoclax | RP-normal-1 | knot=0, gamma0=-0.7505429, gamma1= 0.5610923 |
| PFS of Ibrutinib + venetoclax | RP-normal-1 | knot=0, gamma0=-0.6284460, gamma1= 0.6147563 |
| OS of Ibrutinib + placebo | RP-odds-1 | knot=0, gamma0=-1.160643, gamma1= 1.004771 |
| PFS of Ibrutinib + placebo | RP-hazard-2 | knot=0, gamma0=-0.8897710, gamma1= 0.8732763 |

Abbreviations: OS: overall survival; PFS: progression-free survival; RP: Royston-Parmar spline models.

# Supplementary Figure

# A

#
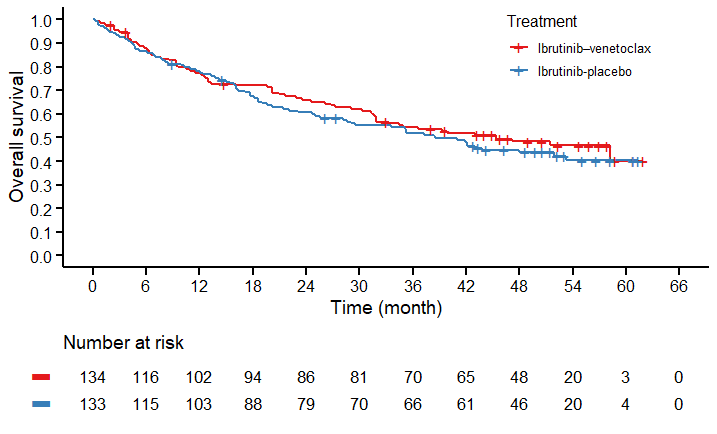


**B**

#
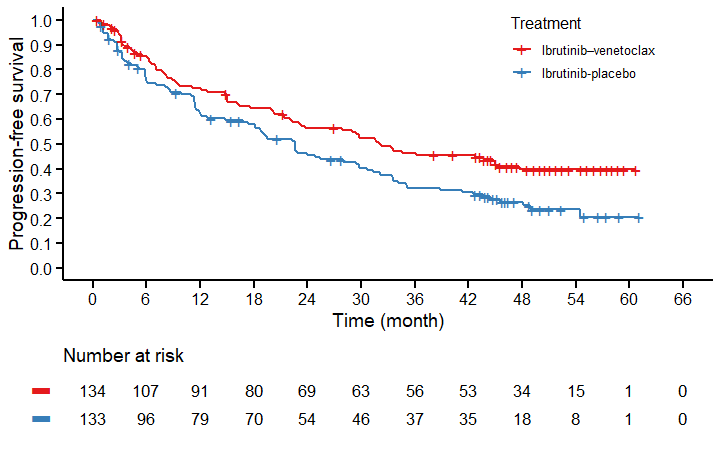


**Supplementary Figure 1.** Reconstruction of Kaplan-Meier survival curves. (A) Overall survival curve. (B) Progression-free survival curve.


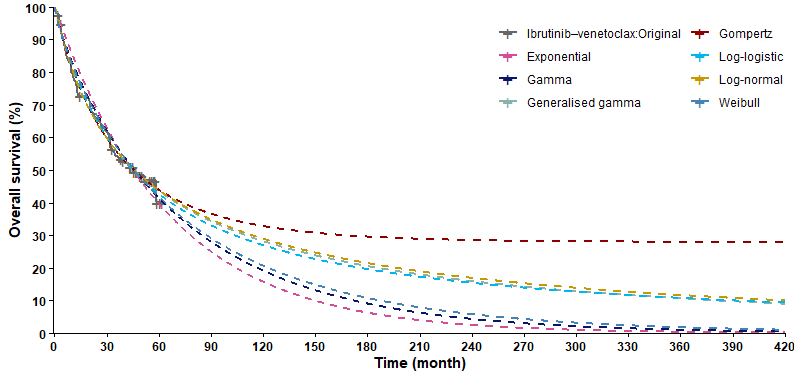


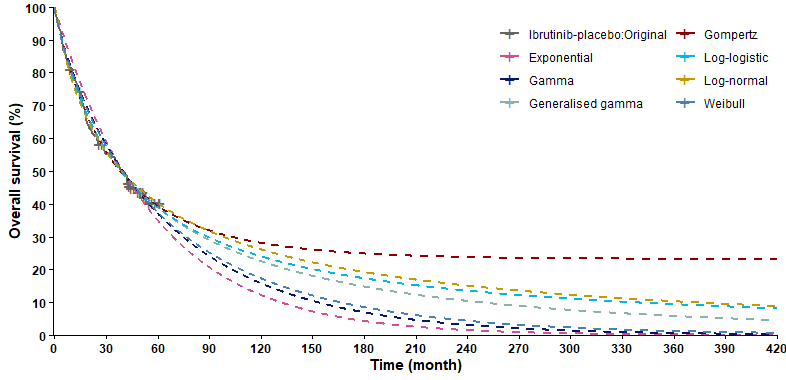


**Supplementary Figure 2.** Progression-free survival curve fitting and extrapolation.


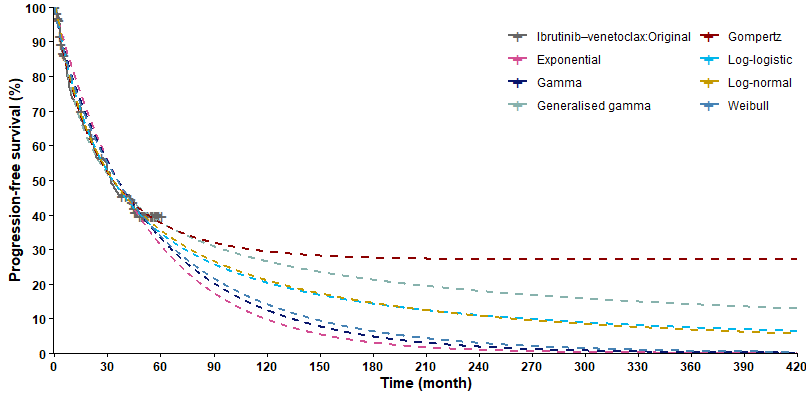


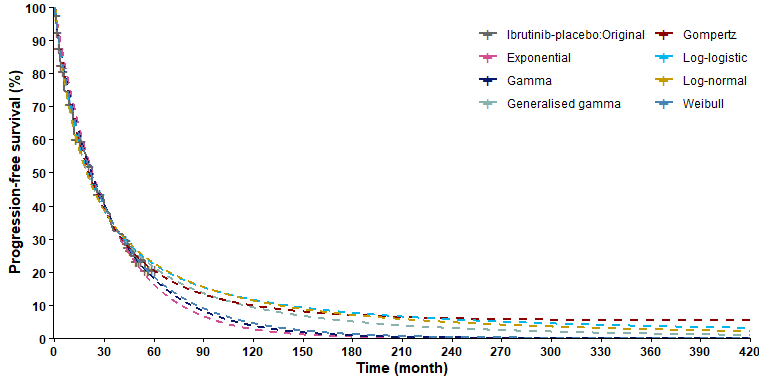


**Supplementary Figure 3.** Overall survival curve fitting and extrapolation.


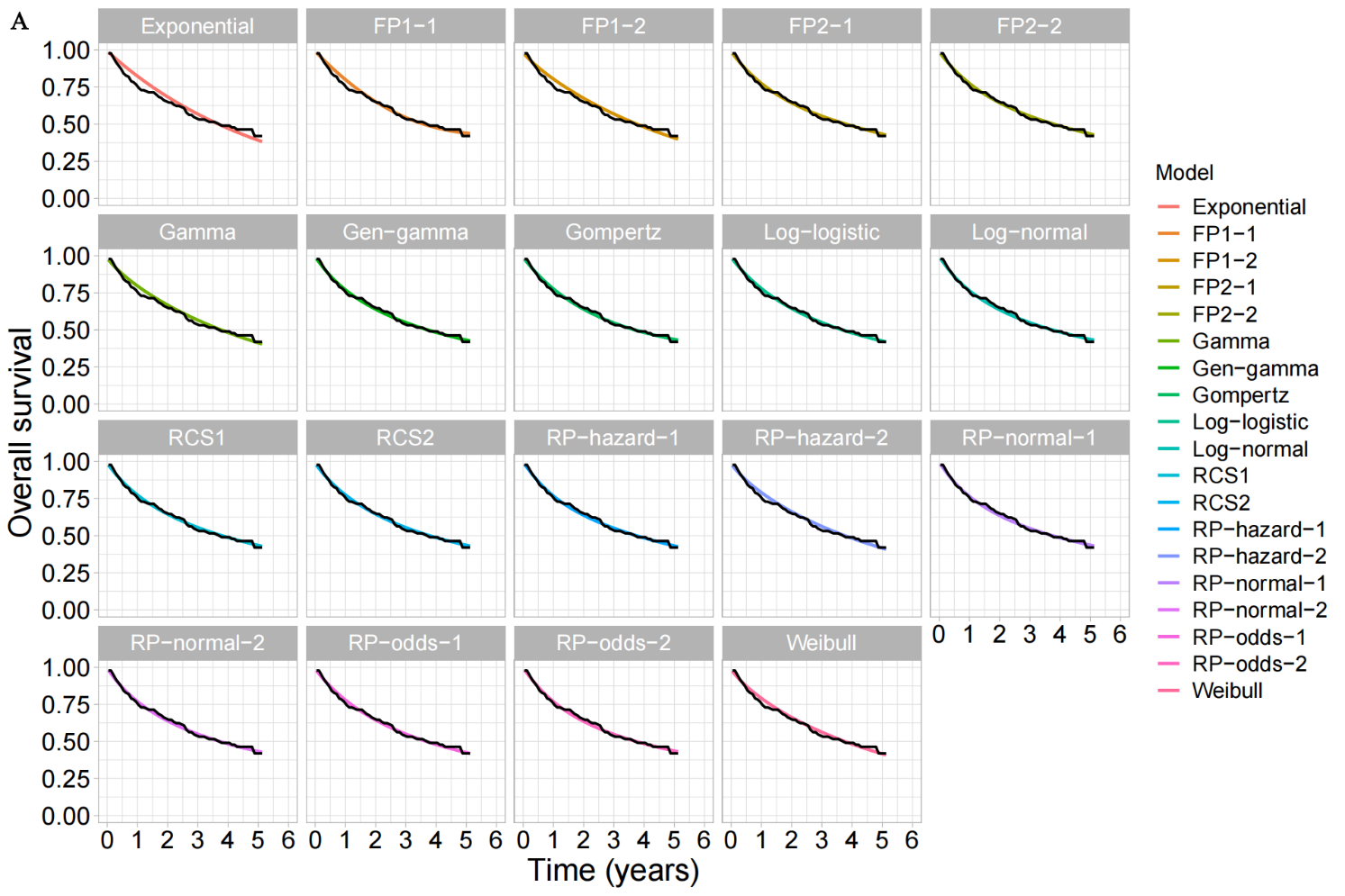

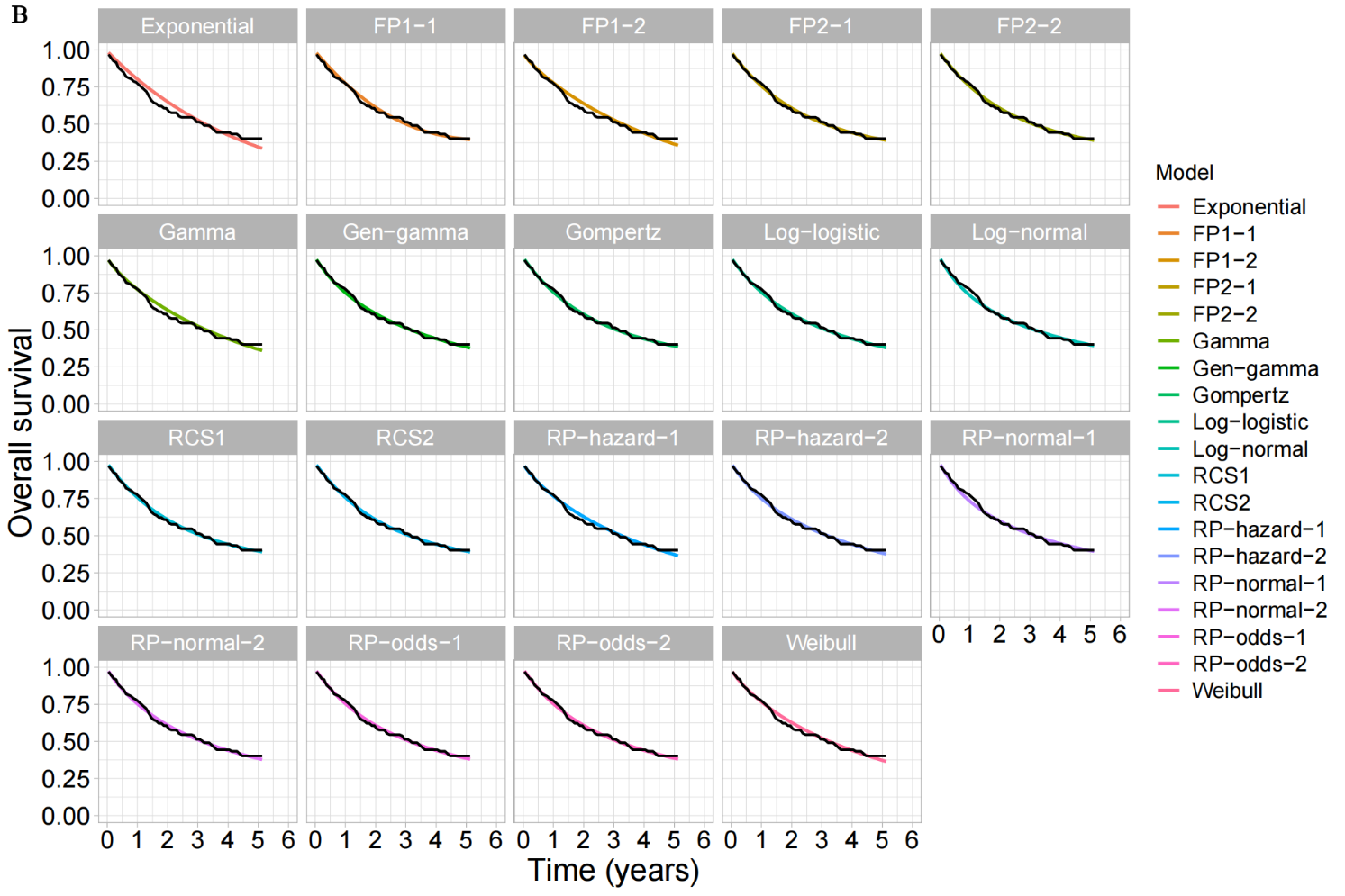


**Supplementary Figure 4.** Overall survival curves plots showing the goodness-of-fit. (A) Ibrutinib + venetoclax group. (B) Ibrutinib + placebo group.

**
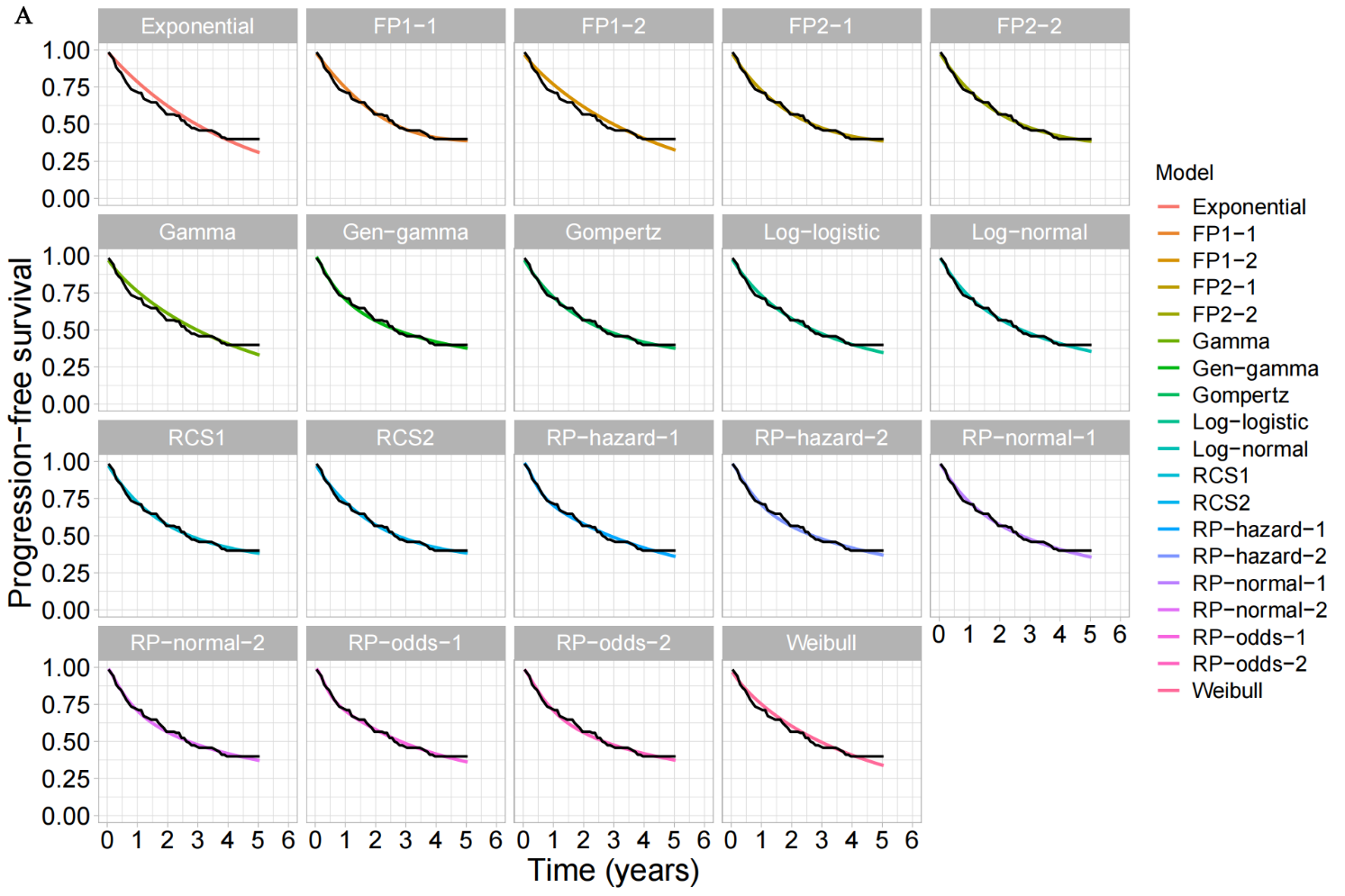
**

**
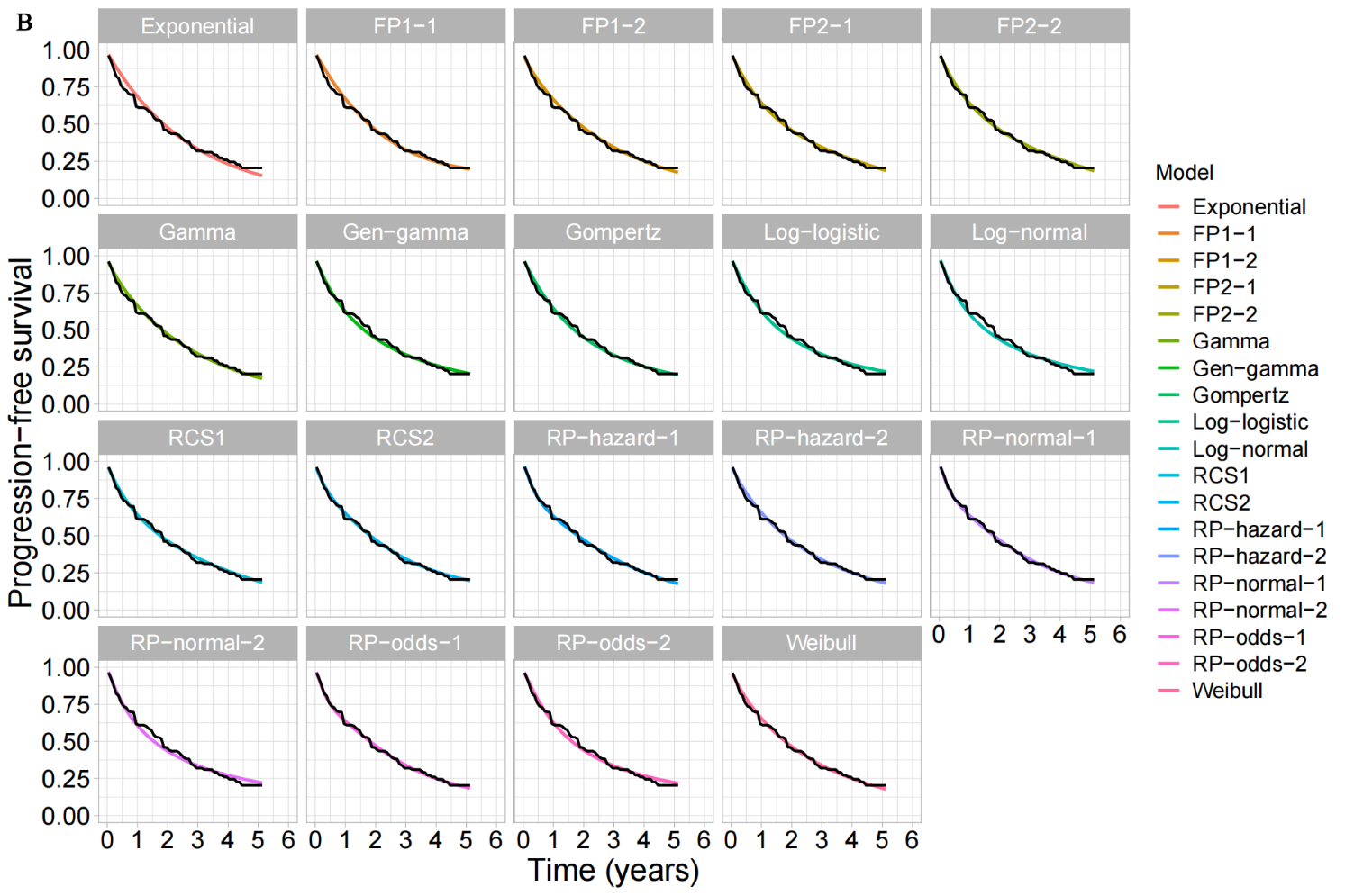
**

**Supplementary Figure 5.** Progression-free survival curves plots showing the goodness-of-fit. (A) Ibrutinib + venetoclax group. (B) Ibrutinib + placebo group.
